# *Streptomyces* sp. BV410 isolate from chamomile rhizosphere soil efficiently produces staurosporine with antifungal and antiangiogenic properties

**DOI:** 10.1002/mbo3.986

**Published:** 2020-01-28

**Authors:** Marija Mojicevic, Paul M. D'Agostino, Aleksandar Pavic, Sandra Vojnovic, Ramsankar Senthamaraikannan, Branka Vasiljevic, Tobias A. M. Gulder, Jasmina Nikodinovic‐Runic

**Affiliations:** ^1^ Institute of Molecular Genetics and Genetic Engineering University of Belgrade Belgrade Serbia; ^2^ Department of Biotechnology and Pharmaceutical Engineering Faculty of Technology University of Novi Sad Novi Sad Serbia; ^3^ Chair of Technical Biochemistry Technische Universität Dresden Dresden Germany; ^4^ Biosystems Chemistry Department of Chemistry and Center for Integrated Protein Science Munich (CIPSM) Technische Universität München Garching bei München Germany; ^5^ AMBER Centre Trinity College Dublin Dublin Ireland

**Keywords:** antiangiogenesis, antifungal activity, secondary metabolite, staurosporine, *Streptomyces* sp., zebrafish

## Abstract

Applying a bioactivity‐guided isolation approach, staurosporine was separated and identified as the active principle in the culture extract of the new isolate *Streptomyces* sp. BV410 collected from the chamomile rhizosphere. The biotechnological production of staurosporine by strain BV410 was optimized to yield 56 mg/L after 14 days of incubation in soy flour–glucose–starch–mannitol‐based fermentation medium (JS). The addition of FeSO_4_ significantly improved the staurosporine yield by 30%, while the addition of ZnSO_4_ significantly reduced staurosporine yield by 62% in comparison with the starting conditions. Although staurosporine was first isolated in 1977 from *Lentzea albida* (now *Streptomyces staurosporeus*) and its potent kinase inhibitory effect has been established, here, the biological activity of this natural product was assessed in depth in vivo using a selection of transgenic zebrafish (*Danio rerio*) models, including *Tg*(*fli1:EGFP*) with green fluorescent protein‐labeled endothelial cells allowing visualization and monitoring of blood vessels. This confirmed a remarkable antiangiogenic activity of the compound at doses of 1 ng/ml (2.14 nmol/L) which is below doses inducing toxic effects (45 ng/ml; 75 nmol/L). A new, efficient producing strain of commercially significant staurosporine has been described along with optimized fermentation conditions, which may lead to optimization of the staurosporine scaffold and its wider applicability.

## Graphical abstract

In this study, Streptomyces sp. BV410, a new, efficient producing strain of commercially significant staurosporine has been described along with optimized fermentation conditions. Staurosporine showed potent antifungal activity against different Candida species as well as potent antiangiogenic activity in zebrafish. This may lead to optimization of the staurosporine scaffold and its wider applicability.

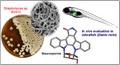

## 1 INTRODUCTION

It has been widely recognized that Actinomycetes, especially members of the genus *Streptomyces*, are one of the most prolific sources of bioactive natural products, including the most important antimicrobial drug classes such as β‐lactams, tetracyclines, rifamycins, polyenes, indocarbazoles, and others (Genilloud, 2017). They also produce various secondary metabolites that are cytotoxic, cytostatic, anti‐inflammatory, antiparasitic, antimalaria, antiviral, antioxidant, and antiangiogenic (Liu, Deng, & Liu, 2018). Frequent rediscovery of the same compounds from the soil isolates has made them less attractive for screening programs in the recent years. Nevertheless, classical screening strategies based on whole cell assays are still successful in discovery of novel bioactive molecules or new biological activities of known compounds from microbial extracts (Donadio, Maffioli, Monciardini, Sosio, & Jabes, 2010; Riahi, Hosni, Raies, & Oliveira, 2019) providing appropriate prioritization approaches (Crüsemann et al., 2017; Xie et al., 2014). For example, the natural product rapamycin (generic name sirolimus), produced by a strain of *S. hygroscopicus*, has been isolated originally as an antifungal agent with excellent activity against *Candida* spp. (Sehgal, 2003); subsequently, its impressive antitumor and immunosuppressive activities have been revealed (Li, Kim, & Blenis, 2014).

Having transitioned from the rare incidence to a serious problem and a leading cause of morbidity in immunocompromised patients, members of the genus *Candida* have recently been added to the list of priority pathogens (McCarthy & Walsh, 2017; Perfect, 2017). Candidiasis is now one of the most frequent hospital‐acquired infections with around 60 000 cases recorded annually (McCarty & Pappas, 2016; Rodloff, Koch, & Schaumann, 2011). Polyene natural products, such as nystatin and amphotericin B, isolated from *Streptomyces nursei* and *S. nodosus*, respectively, represent the oldest family of antifungal drugs and are still useful in the treatment of invasive fungal infections (Chandrasekar, 2011). However, the need for new antifungal drugs is evident, as resistance against polyenes have been on the rise (Dalhoff, 2018). As a part of our effort to identify new antifungal compounds, we have introduced other species of *Candida* (*C. krusei*, *C. parapsilosis* and *C. glabrata*) to complement the standard *C. albicans* in the functional antifungal screen of *Streptomyces* culture extracts (Mojićević et al., 2019), reasoning that following this approach will increase the chances to discover novel lead structures. Herein, we investigated *Streptomyces* sp. BV410, a soil isolate associated with the rhizosphere of chamomile, which was selected for further chemical characterization and investigation due to the antifungal properties of the crude culture extract (minimal inhibitory concentration (MIC) against *C. albicans* of 8 µg/ml).

## 2 MATERIALS AND METHODS

### 2.1 Microbial strain isolation and identification

*Streptomyces* sp. BV410 was isolated from a soil sample associated with rhizosphere of *Matricaria chamomilla* collected in Serbia in 2016 using conditions favouring the growth of sporulating *Actinomyces* (Mojićević et al., 2019). The strain has been cultivated in tryptone soy broth (Difco) at 30°C for 4 days shaking at 180 rpm for DNA isolation (Nikodinovic, Barrow, & Chuck, 2003). Strain has been identified by 16S rDNA sequence analysis using universal bacterial primer set: 27F (5′‐AGAGTTTGATCCTGGCTCAG‐3′) and 1492R (5′‐GGTTACCTTGTTACGACTT‐3′) and the sequence has been deposited under GenBank accession number: MH128156 (Mojićević et al., 2019). The strain has been deposited at the Institute of Soil Science culture collection ISS WDCM375 under accession number ISS625. The phylogenetic tree was constructed by the maximum likelihood algorithm using Jukes–Cantor distance correction and bootstrap resampling method, all included in the MEGA7 package (Kumar, Stecher, & Tamura, 2016). The tree was rooted using the 16S rRNA gene sequence of *Bacillus subtilis* MF993342.1 as an outgroup. Sequences of the nearest type strains, as well as the outgroup strain, were obtained from the GenBank database.

The strain was grown on mannitol–soy flour (MSF; mannitol 20 g/L, soybean flour 20 g/L, agar 20 g/L) agar that was found to promote good growth and sporulation of a majority of *Streptomyces* spp. from laboratory culture collection at 30°C for 4–14 days. Spore suspension of *Streptomyces* sp. BV410 was stored in glycerol (20%, v/v), maintained at −80°C, and used for the inoculation of cultures for further experiments.

### 2.2 Scanning electron microscopy (SEM)

In order to assess the surface morphological characteristics of the hyphae and spores of BV410 isolate, scanning electron microscope was used. Scanning electron micrographs of BV410 grown on MSF agar were obtained by a high‐resolution field emission Zeiss Ultra Plus‐*SEM* (Carl Zesis AG) using InLens detector with an accelerating voltage of 5 kV at working distance of 5 mm. Prior to imaging, BV410 were fixed on to the SEM stubs using carbon tape and sputtered with gold/palladium (80/20 ratio) for 10 s.

### 2.3 Cultivation of *Streptomyces* sp. BV410 and preparation of crude culture extracts

Spore suspensions (20 µl) of S*treptomyces* sp. BV410 were first inoculated into vegetative medium (maltose 15 g/L, tryptone soya broth 8 g/L, yeast extract 4 g/L, CaCO_3_ 2 g/L) and incubated at 30°C for 48 hr, 180 rpm. This preculture was used for inoculation (1%, v/v) of routinely used JS medium (glucose 20 g/L, starch 20 g/L, mannitol 15 g/L, soybean flour 30 g/L, CaCO_3_ 10 g/L). Both of these media were optimized from our previous work with *Streptomyces* spp. and have been described earlier (Stankovic et al., 2013).

Cultures were grown in Erlenmeyer flasks (1:5, culture to volume ratio) containing coiled stainless steel springs for better aeration at 30°C, 180 rpm for 7 and 14 days. Extraction of whole cultures with ethyl acetate (EtOAc; 1:1/v:v), as solvent that would extract a wide range of small molecules of differing polarity (from nonpolar to polar), was performed by vigorous mixing at 30°C for 12 hr. The EtOAc extract was separated from the cell debris and the aqueous phase by centrifugation (4,000 g for 20 min at 4°C; Eppendorf 5804R benchtop centrifuge). The mycelium residue was afterward extracted with methanol (MeOH) (1/10 of the original culture volume) by vigorous mixing at 30°C for 30 min. The MeOH extract was separated from the cell debris by centrifugation (4,000 g for 20 min at 4°C; Eppendorf 5804R benchtop centrifuge). Both extracts were then separately dried with anhydrous MgSO_4_, followed by drying under vacuum (BUCHI Rotavapor^®^ R‐300, Germany), and the dry mass of extracts was determined.

#### 2.3.1 Optimization of culture conditions

The effect of substituting soy flour from the JS medium with yeast extract (3%, w/v; JSYE) and the addition of KH_2_PO_4_ (1%, w/v; JSYEP) as well as two additional media TSB (20 g/L) and R2YE (Kieser, Bibb, Buttner, Chater, & Hopwood, 2000) were assessed as production media. The initial pH of JS medium was adjusted to pH of 6.5, 7.5 and 8.5 using 0.1 mol/L HCl or NaOH. The addition of methyl oleate (Sigma‐Aldrich, 2 ml/L), ZnSO_4_·7H_2_O (0.5 g/L) and FeSO_4_·7H_2_O (0.5 g/L) to JS medium (with adjusting initial pH to 8.5) was also examined.

### 2.4 Isolation, purification, and characterization of the active compound

The 2 g of ethyl acetate extract was completely resuspended in H_2_O (40 ml). This H_2_O (re)suspension was re‐extracted using an equal volume of heptane (excluded to remove nonpolar compounds) for further purification, followed be re‐extraction with EtOAc (using two times equal volume of solvent). The EtOAc and aqueous phases were dried under vacuum and then dissolved in 5 ml of MeOH, followed by separation by size‐exclusion chromatography on Sephadex LH20 (Sigma) with methanol as the mobile phase. Fractions found to contain our molecule of interest (based on activity screening) were pooled and concentrated under vacuum prior to final purification by preparative HPLC. The isolated molecule was used for downstream structure elucidation by NMR and for biological activity assays. Commercially available staurosporine (Fisher BioReagents, Fisher Scientific) was used as an analytical standard (Figure S1 in Appendix S1).

#### 2.4.1 Analytical and semi‐preparative high‐performance liquid chromatography

Dried extracts were dissolved in MeOH and analyzed by analytical HPLC on a JASCO system consisting of a UV‐1575 Intelligent UV/VIS Detector, DG‐2080‐53 3‐Line Degasser, two PU‐1580 Intelligent HPLC Pumps, AS‐1550 Intelligent Sampler, and HG‐1580‐32 Dynamic Mixer controlled by the Galaxie chromatography software (Version 1.8.6.1) provided by Jasco. Chromatographic separation was performed at 25°C on a Eurosphere II 100‐3 C18A (150 × 4.6 mm) column with integrated precolumn manufactured by Knauer. A wavelength of 210 nm was used for detection and a PDA UV spectrum of 200–600 nm over the entire run. Solvents used for chromatographic separation were water (A) and acetonitrile (B) as eluents, both supplemented with 0.05% trifluoroacetic acid. The gradient was set as follows: Preconditioning 5% B (2.0 min); 5% B (0 min) →100% B (45 min) →100% B (54 min) →5% B (59 min), at a constant flow rate of 1 ml/min.

The isolation of the compound of interest was performed on a semi‐preparative HPLC controlled by a Jasco HPLC system consisting of an UV‐1575 Intelligent UV/VIS Detector, two PU‐2068 Intelligent prep. Pumps, a MIKA 1,000 Dynamic Mixing Chamber (1,000 μl Portmann Instruments AG Biel‐Benken), a LC‐NetII/ ADC, and a Rheodyne injection valve. The system was controlled by the Galaxie software. Chromatographic separation was performed on a Eurosphere II 100‐5 C18 A (250 × 16 mm) column with precolumn (30 × 16 mm) provided by Knauer and the solvents used were water (A) and acetonitrile (B) as eluents, both supplemented with 0.05% trifluoroacetic acid. The separation method was performed as follows. Preconditioning 5% B (5.0 min); 5% B (2 min) →55% B (30 min) →100% B (31 min) →100% B (38 min) →equilibrate at 5% B for 7 min, at a constant flow rate of 10 ml/min.

#### 2.4.2 Liquid chromatography–mass spectrometry, high‐resolution ESI mass spectrometry and NMR analysis

Samples were analyzed by LCMS on an UltiMate 3,000 LC System coupled to a LCQ Fleet Ion Trap Mass Spectrometer (Thermo Scientific). Chromatographic separation was performed on a Hypersil Gold aQ C18 column (150 × 2.1 mm, 3 µm particle size). Water (A) and acetonitrile (B) were used as the eluents, both supplemented with 0.1% formic acid. The separation method was performed at 0.7 ml/min using a gradient as follows: 5% B at 0 min to 95% B by 8 min followed by washing the column at 100% B for 2 min and re‐equilibration of the column at 5% B for 2 min prior to the next injection. HR‐ESI‐MS spectra were recorded with a Thermo LTQ‐FT Ultra coupled with a Dionex UltiMate 3,000 HPLC system.

NMR spectra were recorded on Bruker AVHD300, Bruker AVHD400, Bruker AVHD500 (only ^1^H NMR spectra), or Bruker AV500‐cryo spectrometers. The chemical shifts are listed as parts per million (ppm) and refer to (TMS; Tetramethylsilane) = 0. The spectra were calibrated using residual undeuterated solvent as an internal reference (CDCl_3_ = 7.26 ppm, (CD_3_)_2_CO = 2.05 ppm, (CD_3_)_2_SO = 2.50 ppm, CD_3_OD = 3.31 for 1H NMR; CDCl3 = 77.0 ppm, (CD_3_)_2_CO = 29.8 ppm, (CD_3_)_2_SO = 39.5 ppm, CD_3_OD = 49.0 for ^13^C NMR).

### 2.5 Anti‐*Candida* assays

Antifungal activity was tested by standard disc diffusion assays against type strains: *C. albicans* ATCC 10231, *C. krusei* ATCC 6258, *C. parapsilosis* ATCC 22019, *C. glabrata* ATCC 2001. These strains are among top five species most commonly associated with candidiasis (Turner & Butler, 2014). MICs were determined for crude BV410 culture extracts and for purified staurosporine according to CLSI broth microdilution guidelines (CLSI M27‐A4, 2012 and CLSI M27‐A3, 2008). MICs were determined in RPMI – 1,640 medium (Sigma, Aldrich) as specified in the standards. The MIC value corresponds to the lowest concentration that inhibited the growth of the respective test organism after 24 hr at 37°C. The highest concentration of the tested compounds used in these assays was 250 µg/ml.

### 2.6 Cytotoxicity assay

Standard colorimetric MTT (3‐[4,5‐dimethylthiazol‐2‐yl]‐2,5‐diphenyltetrazolium bromide) assay with MRC5 (human healthy fibroblasts as model of healthy cell line) and A549 (human epithelial adenocarcinoma as corresponding model of cancer cell line) was performed (Hansen, Nielsen, & Berg, 1989). Briefly, cells were plated in a 96‐well flat‐bottom plate at a concentration of 1 × 10^5^ cells per well and maintained as monolayer cultures in RPMI‐1640 medium. Isolated staurosporine was dissolved in DMSO and filter sterilized (0.2 µm, EMD Millipore) to prepare a stock solution (50 mg/ml) and added to the cells at a concentration of 0.78–50 ng/ml for a treatment that lasted for 48 hr. Results were presented as percentage of the DMSO‐treated control that was set to 100%. The percentage viability values were plotted against the log of concentration, and a sigmoidal dose–response curve was calculated by nonlinear regression analysis using the GraphPad Prism software, version 5.0 for Windows (GraphPad Software). Cytotoxicity was expressed as the concentration of the compound inhibiting growth by 50% (IC_50_).

### 2.7 In vivo embryotoxicity assay

The in vivo toxicity assessment of isolated staurosporine was carried out in the zebrafish *(Danio rerio*) model and in compliance with the European directive 2010/63/EU and the ethical guidelines of the Guide for Care and Use of Laboratory Animals of the Institute of Molecular Genetics and Genetic Engineering, University of Belgrade. The effect of staurosporine on the zebrafish embryos survival and development was examined according to the OECD 2013 guidelines for the testing of chemicals (OECD, 2013) and following previously described protocol (Warżajtis et al., 2017) with some modifications. Briefly, zebrafish embryos were produced by pair‐wise mating of wild‐type adults, collected and distributed into 24‐well plates containing 10 embryos per well and 1 ml embryos water (0.2 g/L of Instant Ocean^®^ Salt in distilled water), and raised at 28°C. For assessing lethal and developmental toxicity, embryos at the 6 hr postfertilization (hpf) stage were treated with eight concentrations of staurosporine (1, 10, 20, 30, 35, 40, 50, and 60 ng/ml). DMSO (0.125%, v v^−1^) was used as a negative control. Experiments were performed in triplicate using 30 embryos per concentration. Apical endpoints used for the toxicity evaluation (Table S1; Appendix S1) were recorded at 24, 48, 72, 96, and 120 hpf using an inverted microscope (CKX41; Olympus). Dead embryos were counted and discarded every 24 hr. At 120 hpf, embryos were inspected for heartbeat rate, anesthetized by addition of 0.1% (w v^−1^) tricaine solution (Sigma‐Aldrich), photographed and killed by freezing at −20°C for ≥24 hr.

The LC_50_ value (the concentration upon which 50% embryos were dead) and the EC_50_ value (the concentration affecting 50% of embryos) were determined by the program ToxRatPro (ToxRat^®^, Software for the Statistical Analysis of Biotests, ToxRat Solution GmbH, Version 2.10.05) using the probit analysis with linear maximum likelihood regression.

#### 2.7.1 Hepatotoxicity and myelotoxicity evaluation in the zebrafish model

In order to examine the isolated staurosporine for a possible hepatotoxic effect in vivo, the transgenic *Tg*(*fabp10*:EGFP) zebrafish embryos with the fluorescently labeled liver were treated at the 72 hpf stage (when the liver is fully functional) with five doses (30, 35, 40, 45, and 50 ng/ml) of the tested compound. DMSO (0.125%, v v^−1^) was used as a negative control. The hepatotoxicity was determined according to the change of liver area index compared to the control group, calculated as the ratio between liver area and embryonic lateral area × 100% (Zhang et al., 2017).

To address the possible myelotoxicity of the isolated staurosporine, transgenic zebrafish embryos *Tg*(*mpx:GFP*) expressing enhanced green fluorescent protein (EGFP) in neutrophils were used. The assay was performed according to the previously described protocol (Veselinović et al., 2017) with slight modifications. Briefly, transgenic embryos were generated by natural spawning of *Tg*(*mpx:GFP*) and wild‐type adults and reared in the fish embryo water at 28°C. At the 6 hpf stage, embryos were exposed to three doses (25, 30, and 35 ng/ml) of staurosporine upon which no embryonic malformations were observed. DMSO (0.125%, v/v) was used as a negative control. The transgenic embryos were inspected at 72 hpf stage under a fluorescence microscope (Olympus BX51, Applied Imaging Corp.) for the neutrophils presence and fluorescence intensity. Neutrophils occurrence (fluorescence) was determined by ImageJ program (NIH public domain software).

#### 2.7.2 Antiangiogenic potential evaluation in the zebrafish model

The antiangiogenic activity of the isolated staurosporine was evaluated using transgenic zebrafish *Tg*(*fli1:EGFP*) embryos with EGFP‐labeled endothelial cells, as was previously described (Pavic et al., 2017). Briefly, transgenic embryos were generated by natural spawning of wild‐type and Tg(*fli1:EGFP*) adults and reared in embryo water at 28°C. At 6 hpf, embryos were exposed to the range of nontoxic staurosporine concentrations and incubated at 28°C. After the treatments, embryos were anesthetized with 0.02% tricaine and subsequently photographed. The development of intersegmental blood vessels (ISVs), dorsal longitudinal anastomotic vessels (DLAVs), and of subintestinal vessel (SIV) plexus was inspected and imaged in embryos at 48 hpf and 72 hpf, respectively, under a fluorescence microscope (Olympus BX51, Applied Imaging Corp.). Sunitinib malate (Suten Pfizer), an antiangiogenic drug of clinical relevance, was used as the positive control (Chimote et al., 2014).

### 2.8 Statistical analysis

The results were expressed as mean values ± standard deviation (*SD*) and analyzed using Student's *t* test at a threshold level of *p* = .05. This analysis was carried out using SPSS 20 (SPSS Inc.) software.

## 3 RESULTS

### 3.1 *Streptomyces* sp. BV410 isolate from the rhizosphere of chamomile

Soil isolate BV410 was associated with rhizosphere of *Matricaria chamomilla*, with considerable antifungal activity observed when ethyl acetate extracts of the whole culture grown in JS medium were tested against *C. albicans*, *C. parapsilosis,* and *C. glabrata* (Mojićević et al., 2019). The extract of BV410 showed a MIC against *C. albicans* of 8 µg/ml and the ability to inhibit formation of biofilm at 125 µg/ml (Mojićević et al., 2019). Therefore, strain BV410 was selected for further characterization and chemical investigation.

**Figure 1:**
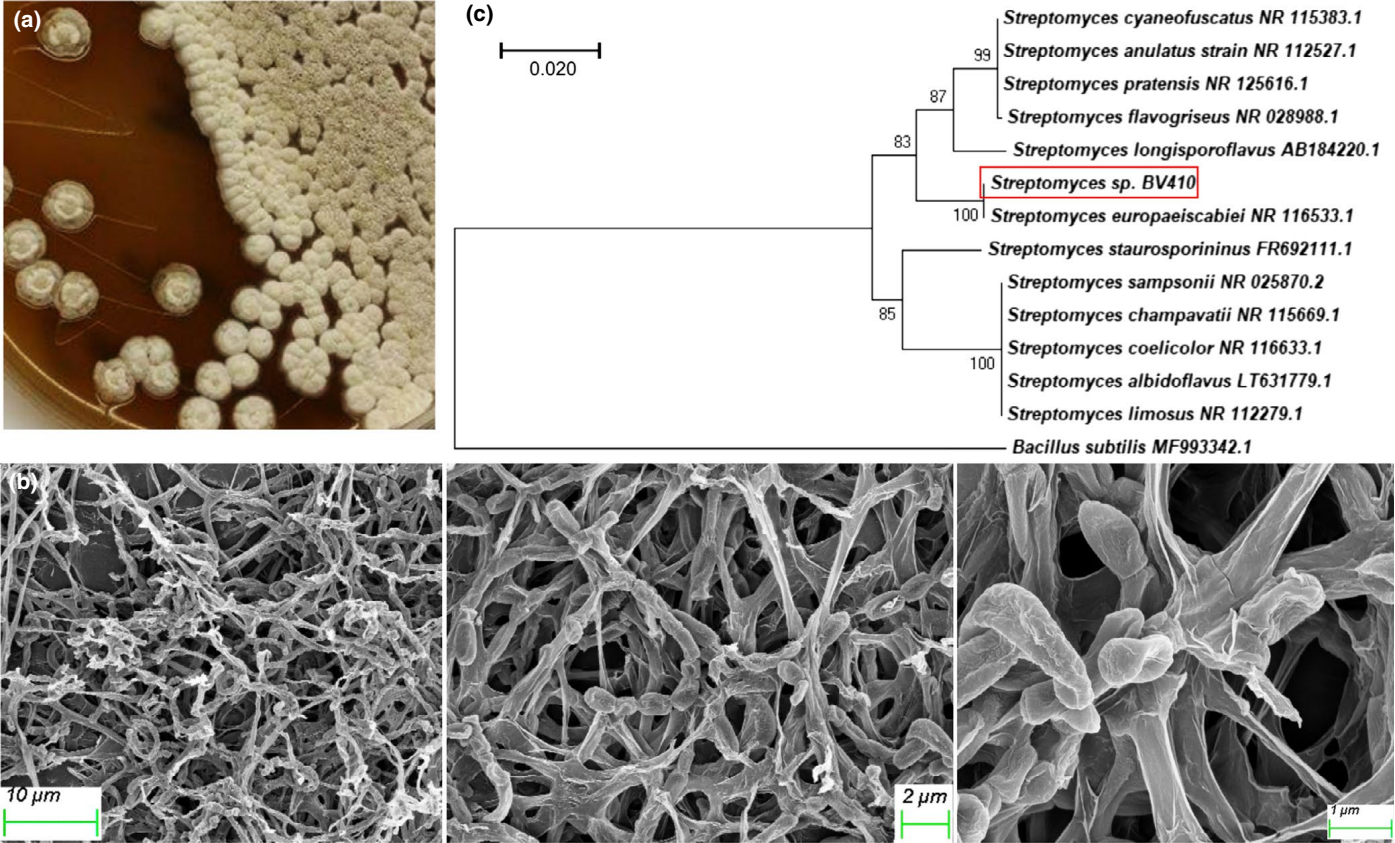
*Streptomyces* sp. BV410. a) Growth and appearance on MSF agar (mannitol 2%, soybean flour 2%, agar 2%) at 30°C for 4 days. b) Scanning electron micrographs of BV410 grown on MSF agar for 7 days at 30°C showing mycelia and spore chains. c) Phylogenetic tree of 12 strains of *Streptomyces* spp. based on the 16S rDNA gene sequence. Sequences of 11 type strains and an outgroup sequence (*Bacillus subtilis* MF993342.1) were obtained from the GenBank. BV410 is marked with a square. The numbers at the branching points are the percentages of occurrence in 1,000 bootstrapped trees. The bar indicates a distance of 0.020 substitutions per site

BV410 grows well on a variety of standard solid media utilized for Streptomycetes, including ISP‐2, TSB, and oatmeal agar (Kieser et al., 2000). We have chosen MSF for general propagation of this strain due to the abundant velvety aerial mycelium white in color within 4 days of incubation at 28°C (Figure 1a). No distinctive pigment was observable on the reverse side of the colonies on this medium (data not shown). Strain BV410 forms well‐developed aerial mycelia with simple branching and hyphae bearing spores (Figure 1b). The observed spores were cylindrical, approximately 1 µm by 0.6 µm with a smooth surface (Figure 1b).

Using 16S rDNA sequence analysis, strain BV410 was confirmed to belong to the genus *Streptomyces* (the sequence has been deposited under GenBank Accession number MH128156). 16S rDNA sequence alignment and phylogenetic analysis revealed closest similarity to *Streptomyces europaeiscabiei* (1,273 nucleotides, 98% query coverage, 99% homology), isolated from potato scab lesions in Korea (Figure 1c) (Song, Lee, Kang, Baek, & Suh, 2004).

### 3.2 Isolation, purification, and characterization of the active compound

**Figure 2:**
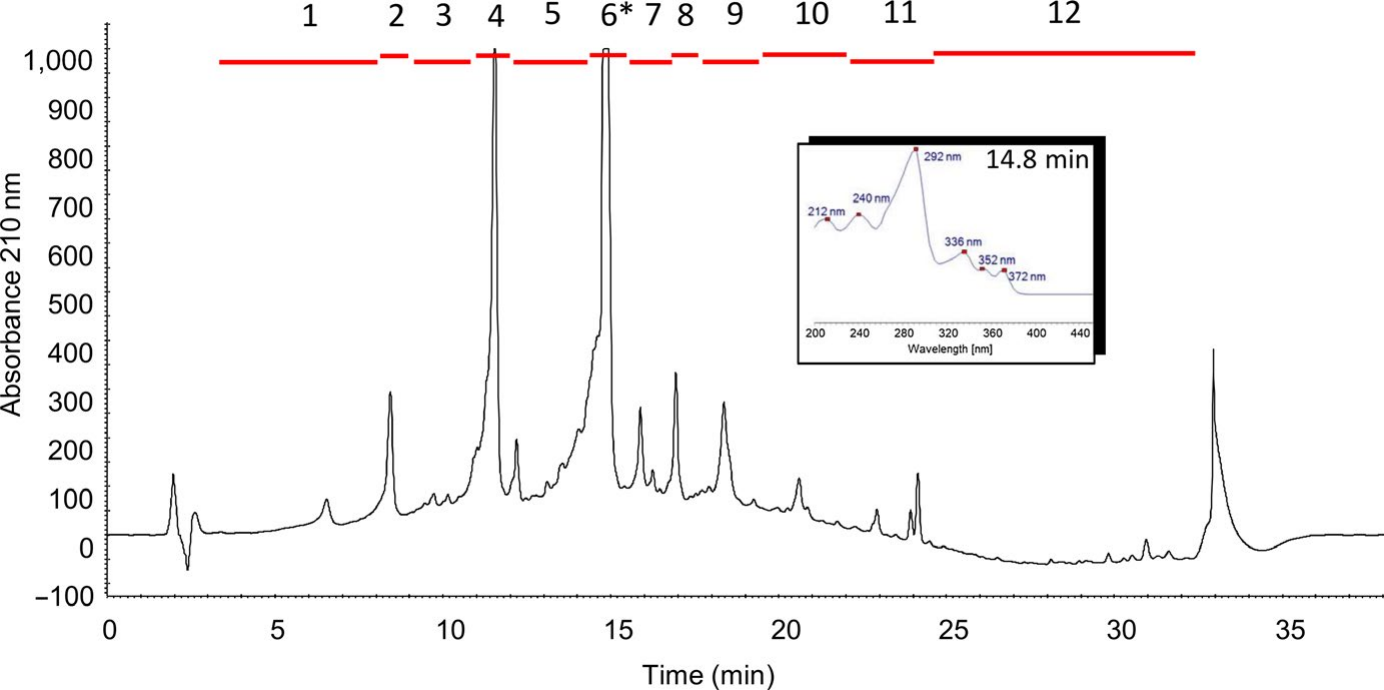
HPLC analysis and fractionation of the BV410 crude extract. The crude extract was collected as multiple fractions (red bars) which were individually tested for antifungal activity. Fraction 6 (*) with a retention time of 14.5–15.5 min showed potent antifungal activity. Fraction 6 was further purified, and a UV absorbance spectrum was obtained (inlet)

Ethyl acetate extracts of BV410 whole culture were analyzed using analytical HPLC and further fractionated by semi‐preparative HPLC. Antifungal bioactivity assays of each fraction revealed the active component as a major compound within fraction 6 (retention time from 14.5 to 15.5 min) of the extract (Figure 2). For preparative compound isolation, the BV410 crude extract was further extracted with solvents ranging in polarity (heptane/ethyl acetate/aqueous). The active compound was predominantly found in the ethyl acetate and aqueous extracts, which was further separated by size‐exclusion chromatography using Sephadex LH‐20 followed by purification using preparative HPLC. The isolated product was analyzed by high‐resolution ESI mass spectrometry by direct injection, revealing a molecular mass of *m/z* 467.2070 [M + H]^+^, and a calculated chemical formula of C_28_H_27_N_4_O_3_ [M + H]^+^. Literature research with this MS data and the calculated molecular formula revealed that the compound might be staurosporine. This was also in line with the observed UV spectrum of the compound (Figure 2, inlet) and was further validated by 1D and 2D NMR analysis (Figure S2 in Appendix S2). Significant differences in ^1^H and ^13^C NMR chemical shifts of our isolated staurosporine sample when compared to most values reported in the literature, particular at the amino sugar portion, could be explained by protonation of the amine function as a consequence of staurosporine isolation using an acidic buffer system. This lead to a major conformational change in this molecular portion with the observed changes in NMR chemical shifts, as previously described in the literature (Link et al., 1996). We furthermore corroborated our NMR structure elucidation results by comparison of our material to an authentic commercial standard by HPLC‐UV‐MS (Figure S3 in Appendix S3). Both compounds indeed perfectly matched (HPLC retention time, UV spectrum, MS spectrum), thus validating the above assignment.

### 3.3 Optimization of staurosporine production by BV410

**Figure 3:**
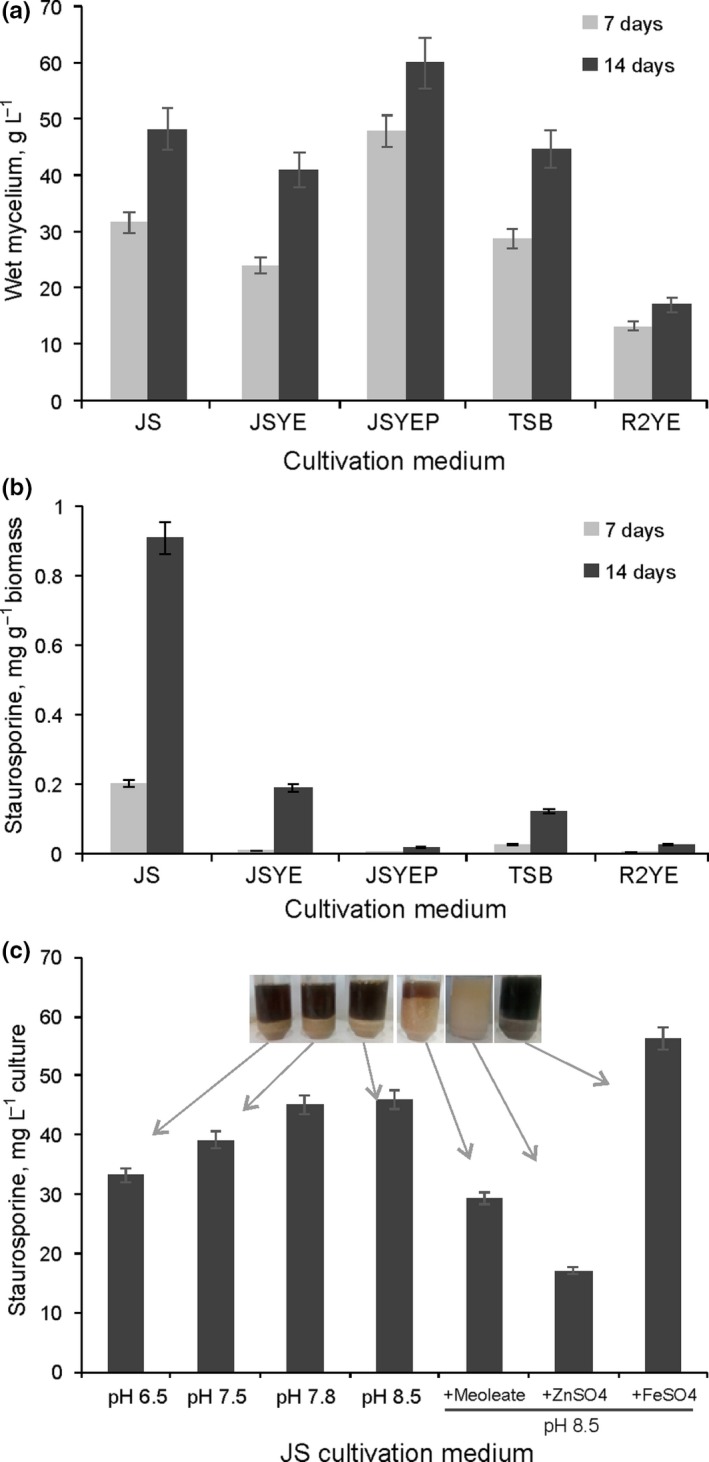
Optimization of staurosporine production in *Streptomyces* sp. BV410 (a) Biomass (wet weight; in grams per liter) and (b) staurosporine (in milligrams per gram of wet mycelia) concentration in cultures grown for 7 (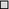) and 14 (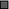) days at 28°C and 200 rpm in five different media (JS, JSYE, JSYEP, TSB, and R2YE). (c) Staurosporine yield (in milligrams per liter of culture) from cultures grown in JS medium for 14 days, with the initial pH values adjusted to 6.5, 7.5, and 8.5 and with the addition of supplements (methyl oleate, ZnSO_4,_ and FeSO_4_)

Having determined that the active secondary metabolite produced by *Streptomyces* sp. BV410 was staurosporine (Figure 2, Figure S2, and Figure S3), we have quantified the staurosporine titer by comparison with the commercial standard to facilitate optimization of the biotechnological production parameters (Figure 3). Firstly, separate extractions of the BV410 culture supernatant and mycelium were carried out to confirm that more than 95% of staurosporine was exported to the medium (data not shown). Then, the effect of substituting soy flour with yeast extract (3%, wv^−1^; JSYE) and the addition of KH_2_PO_4_ (1%, wv^−1^; JSYEP) to the JS medium (glucose 2%, starch 2%, soy flour 2%, mannitol 1.5%, CaCO_3_ 1%) as well as two additional media TSB and R2YE were assessed as production media (Figure 3). The medium supporting the highest biomass yield was JSYEP, with 47.8 g of wet mycelia per liter after 7 days, which was comparable to biomass of 48.2 g/L obtained in JS for 14 days (Figure 3a). The defined R2YE medium supported the lowest growth after both 7 and 14 days of incubation, approximately 3.5‐fold lower in comparison with JSYEP. Despite the pronounced effects of JSYEP in supporting growth of strain BV410, staurosporine yield was the highest in the complex JS medium 0.9 mg/g (45 mg/L) after 14 days of incubation. This was 6.8‐fold higher in comparison with the amount of staurosporine detected after 7 days of incubation in the same medium (Figure 3b). The same trend of considerably higher amount of staurosporine after 14 days was detected in all tested media. Despite the high amount of biomass, the yield of staurosporine from JSYEP medium was quite poor 0.36 ng/g (0.2 mg/L).

Having established that JS was optimal for the staurosporine production over 14 days, the initial pH values were adjusted to 6.5, 7.5, and 8.5 with the unadjusted pH value of the JS medium being 7.8 (Figure 3c). Lowering the initial pH value of the production medium turned out to be suboptimal for the final staurosporine yield (reduction of 13% and 26% has been observed), while the increase to pH 8.5 resulted in the slight increase in the staurosporine yield (2%–5%). The addition of methyl oleate, ZnSO_4,_ and FeSO_4_ to JS medium with the initial pH adjusted to 8.5 on the staurosporine yield was also assessed (Figure 3c). The presence of methyl oleate and ZnSO_4_ resulted in the 35% and 62% reduction, respectively, while the addition of FeSO_4_ caused 30% increase in staurosporine amount. Alternatively, methyl oleate and especially ZnSO_4_ had beneficial effect on the biomass yield (Figure 3c inlet). Overall, this initial optimization of the staurosporine production resulted in the defining of the stable fermentation medium and protocol yielding 56.25 mg/L of staurosporine.

### 3.4 Biological activity evaluation of the isolated staurosporine

We next comprehensively evaluated the anti‐*Candida* activity of isolated staurosporine, in vitro cytotoxicity against two cell lines (healthy MRC5 and cancer cell line A549) as well as the in vivo embryotoxicity and antiangiogenic properties in zebrafish (Table 1). Isolated staurosporine inhibited all four *Candida* strains (MIC values between 24 and 390 ng/ml) although the crude extract of BV410 was not active against *C. krusei*. The most sensitive test strain was *C. glabrata* with MIC values between 4‐ and 16‐fold lower in comparison with the other three *Candida* spp. In a comparison, under the same conditions MIC values for standard antifungal nystatin were between 0.125 and 2 µg/ml (Table S2).

**Table 1:** The biological activity of isolated staurosporine given as MIC values against *Candida* spp., IC_50_ against MRC5 and A549 cell lines, LC_50_ against zebrafish and the lowest antiangiogenesis dose is indicated in ng/ml and nM

Assay	ng/ml	nM
*C. albicans* a	98	210
*C. krusei* a	390	835
*C. parapsilosis* a	98	210
*C. glabrata* a	24	51
MRC5 (healthy fibroblasts)^b^	2	4
A549 (lung carcinoma)^b^	3	6
Zebrafish^c^	45	91
Angiogenesis effective dose	1	2

a MIC, minimal inhibitory concentration.

b IC_50_, the concentration inducing the death of 50% cells.

c LC_50,_ the concentration inducing the lethal effect of 50% embryos.

**Figure 4:**
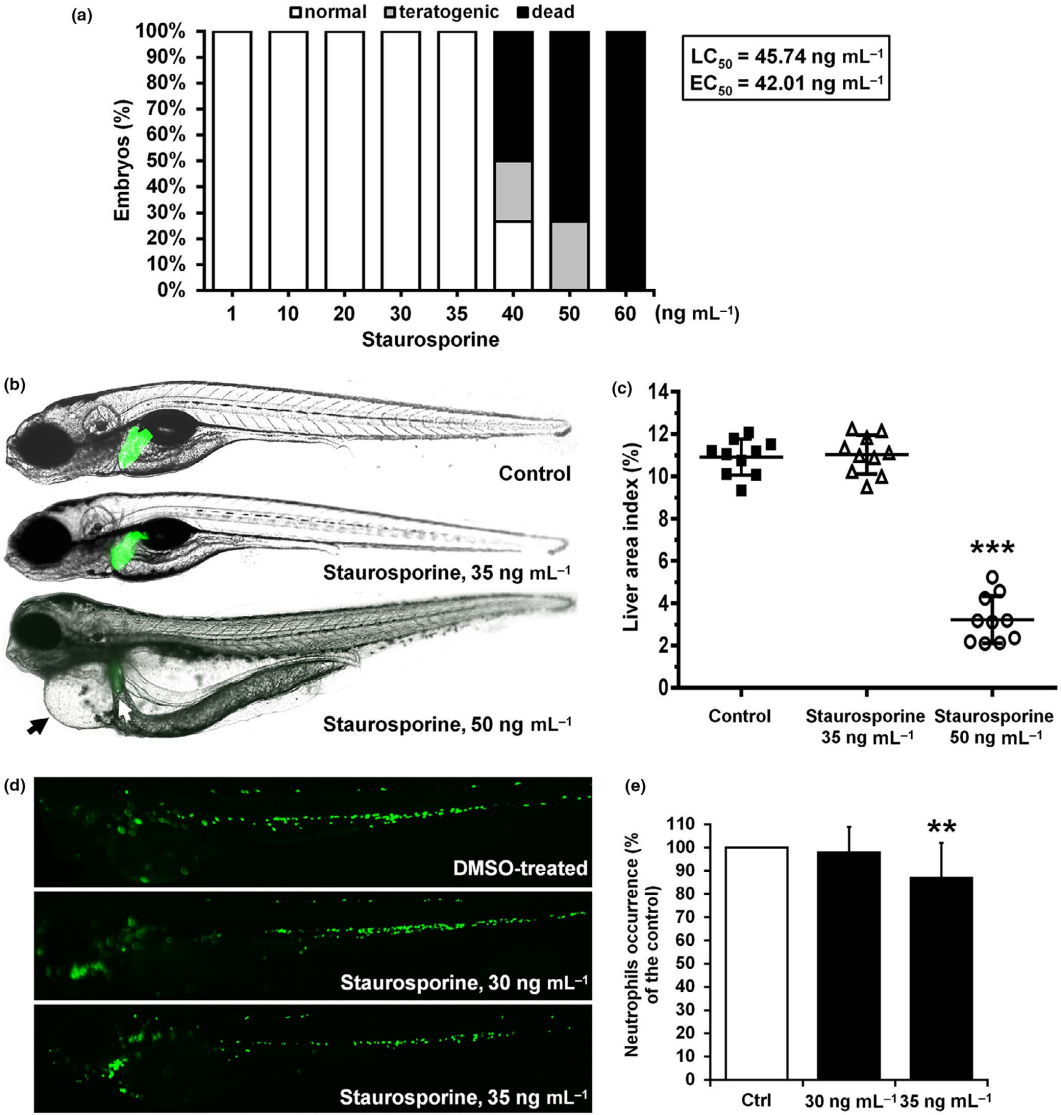
Toxicity assessment of staurosporine in the zebrafish model. (a) The dose‐dependent survival/teratogenicity, (b, c) cardio‐ and hepatotoxicity, and (d, e) myelotoxicity are shown. At a staurosporine dose of 50 ng/ml, embryos were seriously affected, showing large pericardial edema (solid arrow), reduced and damaged liver (white arrow) and whole‐body edema (b). The changes in the liver area index assessed in 120‐hr old zebrafish embryos were not observed between DMSO‐treated (Control) and staurosporine‐treated embryos (35 ng/ml), contrary to the group upon a dose of 50 ng/ml (c). Staurosporine was not myelotoxic at doses up to 35 ng/ml, while at 35 ng/ml, it caused weak neutropenia in the treated zebrafish embryos, as detected at 72 hpf. *p < .05, **p < .01, ***p < .0001

Staurosporine was cytotoxic in vitro to both cell lines at a comparable level (2 and 3 ng/ml), while in vivo toxicity assessment in zebrafish revealed no signs of toxicity at 35 ng/ml (LC_50_ value of 45 ng/ml) (Table 1, Figure 4). Taken together, our results indicated only limited potential of staurosporine as an antifungal agent, but encouraged further evaluation of the compound in the zebrafish model, as zebrafish embryos develop rapidly and are highly accessible for direct microscopic observation.

Zebrafish embryos were exposed to 12 different concentrations (between 1 and 100 ng/ml) of isolated staurosporine up to 120 hpf, and its overall toxicity as well as hepatotoxicity were evaluated (Figure 4). Staurosporine did not cause any observable embryos malformations up to a concentration of 35 ng/ml, while at a dose of 40 ng/ml 50% of the embryos were dead, 23% appeared to be teratogenic (nonresorbed yolk and scoliosis), while 27% of the embryos were without visible deformations (Figure 4a).

Potential cardiotoxicity and liver toxicity of staurosporine were assessed daily from 72 to 120 hpf as these present common drawbacks of drugs approved for human use. The results showed that no embryos exhibited signs of cardiotoxicity at doses ≤35 ng/ml, such as an appearance of pericardial edema, changed heart morphology (Figure 4b), nor disturbed heartbeat rates (data not shown). At doses ≥35 ng/ml, staurosporine induced teratogenic malformations, such as scoliosis (at ≥40 ng/ml), pericardial and whole‐body edema (at ≥45 ng/ml). The transgenic *Tg*(*fabp10*:EGFP) zebrafish embryos with fluorescently labeled liver was used to evaluate the potential hepatotoxicity. The hepatotoxicity was evaluated at 120 hpf old embryos, according to the liver area index (the ratio between liver area and lateral body area) which was shown to be an adequate measure to assess liver damage (Zhang et al., 2017). As shown in Figure 4b,c, staurosporine applied at doses up to 35 ng/ml was not hepatotoxic. However, embryos treated with 50 ng/ml of staurosporine had significantly smaller liver (Figure 4b,c). The transgenic *Tg*(*mpx*:GFP) zebrafish embryos with fluorescently labeled neutrophils were used to assess possible myelosuppression (Figure 4d,e). The obtained results revealed that staurosporine was not toxic toward neutrophils at doses up to 30 ng/ml, but caused slight decrease in their number (13 ± 4% in some embryos at 35 ng/ml) (Figure 4d,e).

**Figure 5:**
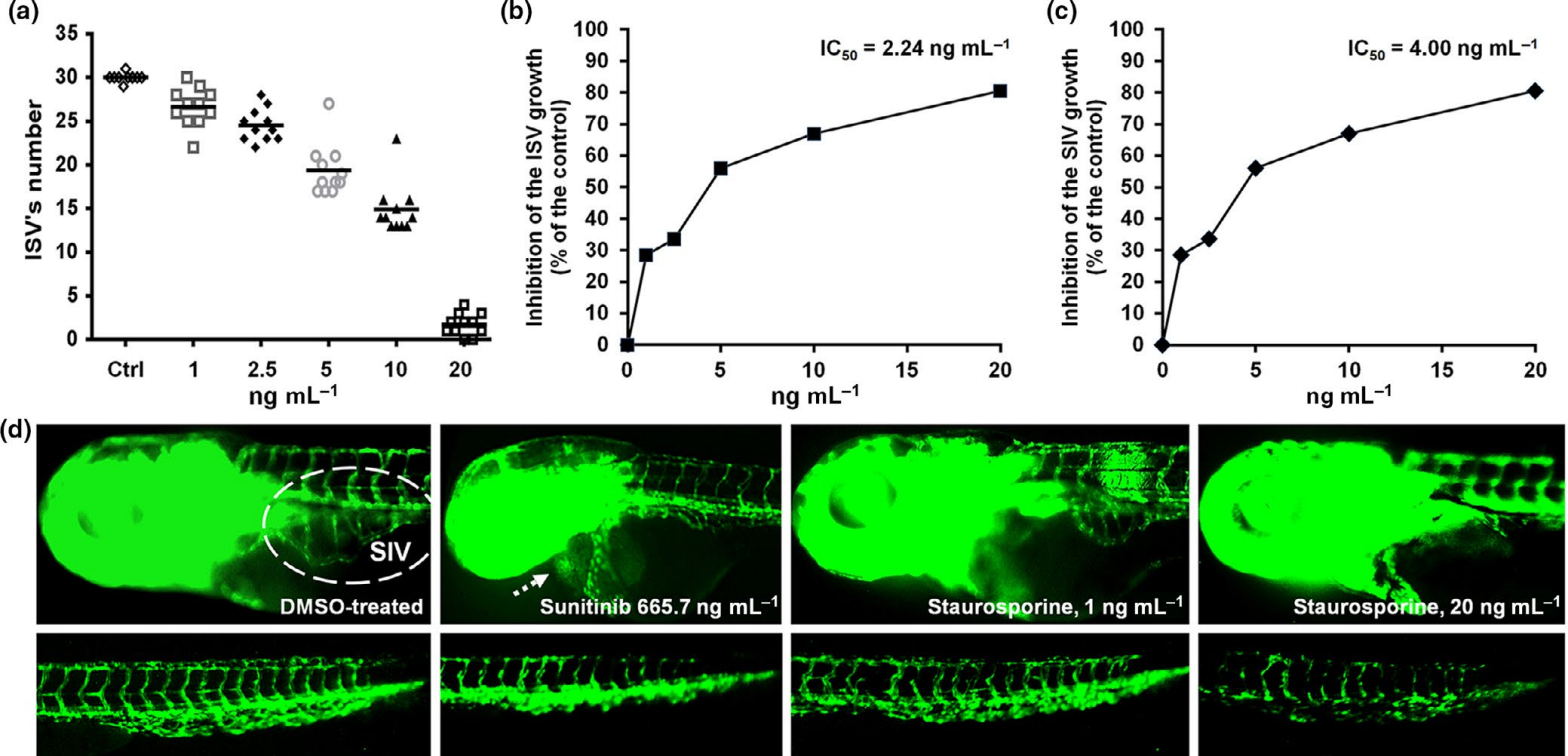
Antiangiogenic activity of staurosporine evaluated in the transgenic *Tg*(*fli1:EGFP*) zebrafish model. A dose‐dependent reduction of a) the number of intact ISV vessels, b) ISV length, and c) SIV basket length. d) Morphology and antiangiogenic phenotype of embryos treated with the antiangiogenic drug sunitinib malate (1.25 µmol/L = 665.7 ng/ml) and 1 ng/ml and 20 ng/ml of staurosporine (2.1 nmol/L and 42.9 nmol/L, respectively)

Some of the embryos treated with 40 ng/ml of staurosporine showed tissue decay and retarded circulation in the tail region, indicating poor angiogenesis. Indeed, results obtained in a transgenic zebrafish line with fluorescently labeled endothelial cells (*Tg*(*fli*:EGFP)) showed that staurosporine inhibited both the intersegmental vessel (ISV) and the subintestinal vessels (SIVs) development in a dose‐dependent manner (Figure 5), exhibiting activity even at a dose of 1 ng/ml (2.14 nM) (Figure S4 in Appendix S4 and Figure S5 in Appendix S5). At a dose of 20 ng/ml (42.8 nM), staurosporine inhibited the development of most of ISV vessels and almost completely blocked SIV basket development. Sunitinib malate (Suten, Pfizer), a clinically used angiogenesis inhibitor, showed comparable activity to staurosporine at 29‐fold higher dose (1.25 µM), also causing an appearance of pericardial edema (Figure 5d) and severe myelosuppression.

## 4 DISCUSSION

Bacterial isolates from rhizosphere soil continue to attract research attention due to plant‐promoting and disease‐controlling activities (Saleh et al., 2019; Thilagam & Hemalatha, 2019). The continuing search for novel antifungal metabolites from bacterial sources carried out in our laboratory led to the isolation of more than 100 rhizosphere isolates associated with ethno‐medicinal plants (poppy, chamomile and nettle) and the evaluation of their crude culture extracts against four *Candida* species (Mojićević et al., 2019). The extract of BV410 had the lowest minimum inhibitory concentration (MIC) value of a total of 412 crude cell extracts screened, with a MIC against *C. albicans* of 8 µg/ml and the ability to inhibit formation of biofilm at 125 µg/ml (Mojićević et al., 2019). Therefore, strain BV410 was selected for further characterization and chemical investigation. Bioactive compound was identified undoubtedly as staurosporine through comprehensive purification and chemical analysis steps (Figure 2, Figure S2, and Figure S3).

The closest similarity of *Streptomyces* sp. BV410 was to *Streptomyces europaeiscabiei*, isolated from potato scab lesions in Korea (Figure 1c) (Song et al., 2004). These two grouped with *S. flavogriseus* and *S. longisporoflavus*, supported by 83% of bootstrap replicates. Interestingly, *Streptomyces* sp. BV410 was not found within the same branch with staurosporine producer *S. staurosporininus* isolated from hay meadow soil from the Cockle Park (Northumberland, United Kingdom) (Kim, Zucchi, Fiedler, & Goodfellow, 2012).

For all bioactive compounds, there is a general interest to obtain high productivities and high titers, as it ensures a cost‐effective production. With Streptomycetes, this has been achieved using classical strain improvement, optimization of process parameters as well as genetic engineering (Kieser et al., 2000; Olmos et al., 2013). The influence of the nutritional parameters on the production of antibiotics is undisputable (Bundale, Begde, Nashikkar, Kadam, & Upadhyay, 2015; Görke & Stülke, 2008). Through the initial optimization of staurosporine production, isolate BV410 was shown to produce 56.25 mg/L of staurosporine (Figure 3). As a comparison, the staurosporine yield of 23 mg/L from a 400‐l fermentation of *Streptomyces* sp. AM‐2282 (the originally described staurosporine producer, now *Lentzea albida* AM‐2282) has been reported after 72 hr cultivation in a medium containing glucose 3%, soybean meal 1.5%, and CaCO_3_ 0.4% for 72 hr (Omura et al., 1977). Several other species of *Streptomyces* have been reported to produce staurosporine and its derivatives with comparable or lower titers (Cheng et al., 2016; Li et al., 2014; Onaka, Taniguchi, Igarashi, & Furumai, 2002; Park et al., 2006; Zhou, Qin, Ding, & Ma, 2018). Staurosporine has also been isolated from the marine ascidian *Cystodytes solitus* (Reyes et al., 2008). Because 56.25 mg/L (0.9 mg/g mycelia) of staurosporine from 400 ml culture in 2 L flask was obtained within this study, future optimization of staurosporine production in BV410 should result in further yield increase. Notably, the addition of exogenous methyl oleate that was previously proposed to cause the alteration in *Streptomyces* spp. membrane permeability and stimulate the accumulation of branched amino acids (Huang, Xia, Li, Wen, & Jia, 2013; Mouslim, David, Pétel, & Gendraud, 1993), negatively affected staurosporine production. Surprisingly, the addition of ZnSO_4_ even more drastically reduced the production of staurosporine in this strain (Figure 3c). This was not in line with the previous findings that both ZnSO_4_ and FeSO_4_ stimulated neomycin production in *Streptomyces fradiae* (Vastrad & Neelagund, 2014).

Because enough pure staurosporine has been isolated, comprehensive biological activity of this molecule was possible in this study. The potent anti‐*Candida* activity is in line with recent reports on staurosporine and analogues to exert activity on virulence and blocking the stress response of *C. albicans* (Baxter, DiDone, Ogu, Schor, & Krysan, 2011; Xie, O'Meara, Polvi, Robbins, & Cowen, 2017). Somewhat higher MIC values were reported against *C. albicans* (6.25 µg/ml) and *C. pseudotropicalis* (3.12 µg/ml) for staurosporine (alkaloid AM‐2282) (Omura et al., 1977) and MIC_80_ of 0.5 µg/ml against *C. albicans* (LaFayette et al., 2010). However, Park et al. suggested that staurosporine isolated from the extraction of *Streptomyces roseoflavus* strain LS‐A24 was not active against *C. albicans*, even at 100 µg/ml, but completely inhibited the mycelial growth of fungal pathogens including *Colletotrichum orbiculare*, *Phytophthora capsici*, *Rhizoctonia solani*, *Botrytis cinerea*, and *Cladosporium cucumerinum* with MIC values of 1–50 µg/ml (Park et al., 2006).

Staurosporine showed anti‐proliferative activity against healthy and cancer cell lines (MRC5 and A549) at a comparable level (2 and 3 ng/ml), which is in line with previous reports on staurosporine nonselective cytotoxicity via potent inhibition of protein kinases, especially tyrosine kinase (Manns et al., 2011; Nakano & Omura, 2009; Tamaoki et al., 1986). On the other hand, in vivo toxicity assessment in zebrafish embryos revealed no signs of toxicity at 35 ng/ml (LC_50_ value of 45 ng/ml) (Table 1, Figure 4). Taken together, our results indicated only limited potential of staurosporine as an antifungal agent, but encouraged further evaluation of the compound in the zebrafish platform. Indeed, using transgenic zebrafish line suitable to monitor angiogenesis, its potent antiangiogenic activity was shown (Figure 5). The antiangiogenic effect of staurosporine was previously studied in a different in vivo assay involving chorioallantoic membranes of growing chick embryos, with the efficient concentrations of 71 pmol per egg (Oikawa et al., 1992). This was followed up with synthesis of a number of staurosporine derivatives that also showed antiangiogenic potential by inhibiting endothelial cell proliferation even in mice (Li et al., 2000; Monnerat et al., 2004). Nowadays, staurosporine is the control agent of choice to induce apoptosis in zebrafish (Eimon & Ashkenazi, 2010).

Our results demonstrate that staurosporine still have high therapeutic potential (therapeutic window, Table S3) especially in the angiogenesis‐related pathologies such as cancer, inflammation, retinopathy, and others. Only tivozanib, vascular endothelial growth factor receptor (VEGFR) inhibitor, has been shown to have better antiangiogenic properties in the zebrafish model (Chimote et al., 2014) compared to staurosporine. Tivozanib was associated with the complete regression in SIVs at 5 nM with acute toxicity in zebrafish at 45 nM and is currently in clinical trials (Chimote et al., 2014).

In this study, although initially aiming at the discovery of novel antifungal compounds against *C. albicans* and non‐*albicans* strains, we have identified a new staurosporine producing *Streptomyces* strain. We have optimized biotechnological production of the compound, which facilitated to assess the toxicity of this known kinase inhibitor in the zebrafish embryo model, including several transgenic lines. This, in turn, confirmed a strong potential of staurosporine as antiangiogenic agent. Recently, staurosporine was shown to potentiate the efficacy of azoles and echinocandins via inhibition of protein kinase C (Pkc1) (LaFayette et al., 2010) thereby helping in evading fungal drug resistance. Furthermore, research has been directed in the analysis of overall toxicity of this compound in vivo, with suggestions that after all, “staurosporine may be granted a new lease of life” for the therapeutic development (Omura, Asami, & Crump, 2018).

## CONFLICT OF INTEREST

None declared.

## AUTHOR CONTRIBUTIONS

Marija Mojicevic: Formal analysis‐Equal, Investigation‐Equal, Writing‐original draft‐Supporting; Paul Michael D'Agostino: Formal analysis‐Equal, Investigation‐Equal, Writing‐original draft‐ Supporting; Aleksandar Pavic: Formal analysis‐Supporting, Investigation‐Supporting, Methodology‐Supporting, Writing‐original draft‐Supporting; Sandra Vojnovic: Conceptualization‐Supporting, Project administration‐Supporting, Supervision‐Equal, Writing‐original draft‐Supporting; Ramsankar Senthamaraikannan: Investigation‐ Supporting, Methodology‐Supporting, Writing‐original draft‐Supporting; Branka Vasiljevic: Funding acquisition Supporting, Project administration‐Supporting, Writing‐review & editing‐Supporting; Tobias Gulder: Conceptualization‐Equal, Formal analysis‐Equal, Funding acquisition‐Equal, Writing‐original draft‐Equal; Jasmina Nikodinovic‐Runic: Conceptualization‐Equal, Formal analysis‐Equal, Funding acquisition‐Equal, Investigation‐Equal, Project administration‐Equal, Writing‐original draft‐Lead.

## ETHICS STATEMENT

Protocols and procedures employed in this investigation were approved by the appropriate institutional committees. Zebrafish (*Danio rerio*) were kept and handled in compliance with the guidelines of the European Union for handling laboratory animals (https://ec.europa.eu/environment/chemicals/lab_animals/index_en.htm). Experiments were terminated before the zebrafish larvae reached the free feeding stage and did not classify as animal experiments according to the 2010/63/EU Directive.

## Figures and Tables

**Figure A1:**
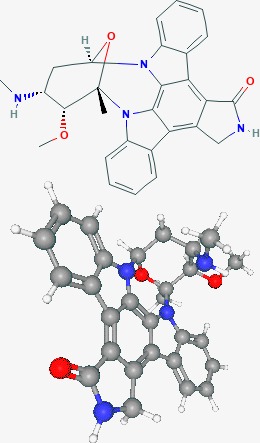
Chemical structure of staurosporine

**Figure A2:**
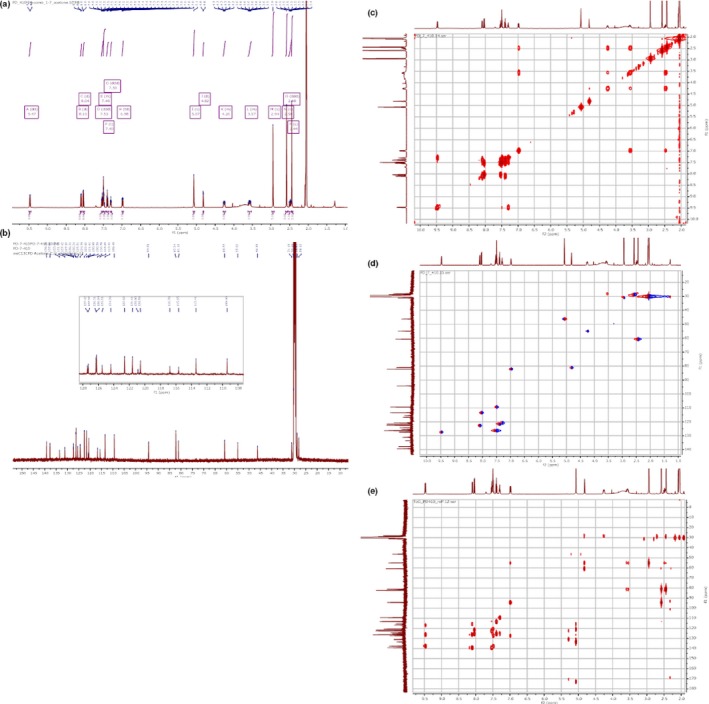
NMR data recorded for isolated staurosporine (acetone‐d6, 500 MHz ^1^H, 125 MHz 13C). (a) ^1^H NMR spectrum of staurosporine; (b) 13C NMR spectrum of isolated staurosporine; (c) 2D COSY spectrum of isolated staurosporine; (d) HSQC spectrum of isolated staurosporine; (e) HMBC spectrum of isolated staurosporine

**Figure A3:**
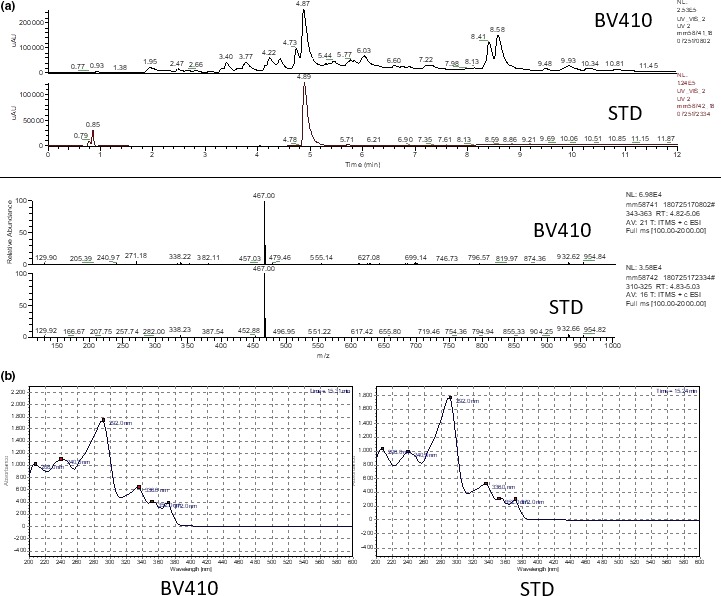
Comparison of BV410 derived staurosporine with the commercially available analytical standard. (a) LCMS results of BV410 raw extract and staurospoine standard. The top panel is UV absorbance chromatogram at 280 nm and the bottom panel is the mass spectrum at retention time at 4.8–5.0 minutes. (b) UV spectrum of BV410 derived staurosporine (left) with the commercially available staurosporine sample obtained using HPLC

**Figure A4:**
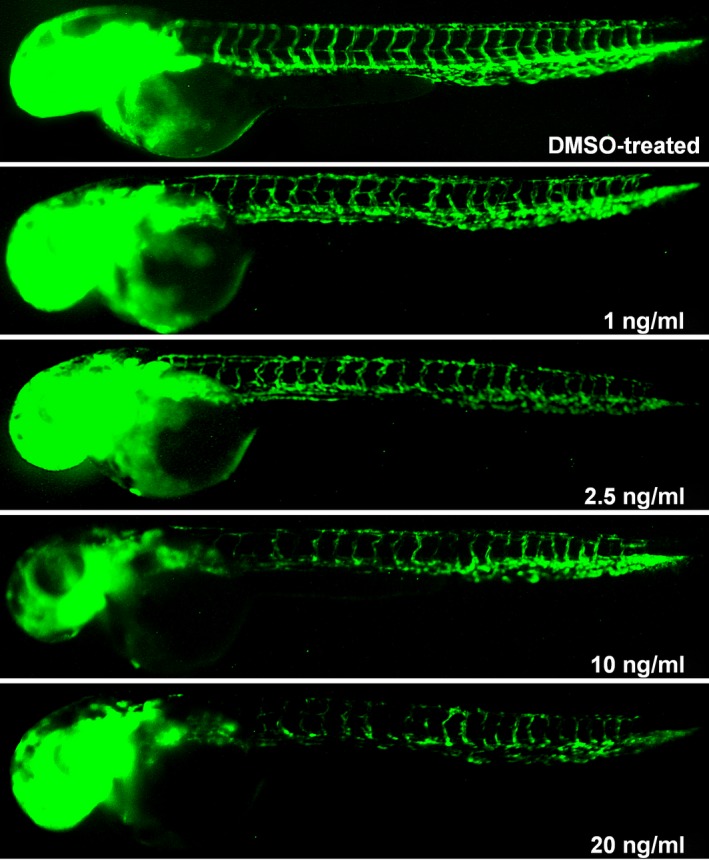
The effect of different staurosporine concentrations on the ISV angiogenesis addressed at 48 hrs post fertilization (hpf)

**Figure A5:**
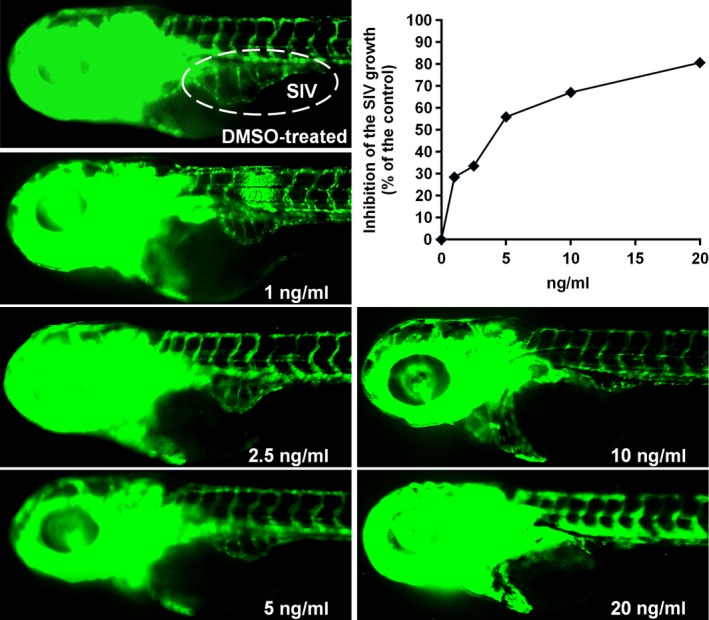
Inhibition of the SIVs angiogenesis in developing zebrafish embryos by different staurosporine concentrations addressed at 72 hpf

## Data Availability

The strain has been deposited at the Institute of Soil Science (Belgrade, Serbia) culture collection ISS WDCM375 (http://www.wfcc.info/ccinfo/collection/by_id/375) under accession number ISS625. Partial sequence of 16S DNA has been deposited under GenBank accession number MH128156 (https://www.ncbi.nlm.nih.gov/nuccore/MH128156).

## APPENDIX 1

**Table A1:** Lethal and teratogenic effects observed in zebrafish (*Danio rerio*) embryos at different hours post fertilization (hpf)

Category	Developmental endpoints	Exposure time (hpf)			
		24	48	72	96/120
Lethal effect	Egg coagulation^a^	●	●	●	●
	No somite formation	●	●	●	●
	Tail not detached	●	●	●	●
	No heart‐beat		●	●	●
Teratogenic effect	Malformation of head	●	●	●	●
	Malformation of eyes^b^	●	●	●	●
	Malformation of sacculi/otoliths^c^	●	●	●	●
	Malformation of chorda	●	●	●	●
	Malformation of tail^d^	●	●	●	●
	Scoliosis	●	●	●	●
	Heart beat frequency		●	●	●
	Blood circulation		●	●	●
	Pericardial edema	●	●	●	●
	Yolk edema	●	●	●	●
	Yolk deformation	●	●	●	●
	Growth retardation^e^	●	●	●	●

a No clear organs structure are recognized.

b Malformation of eyes was recorded for the retardation in eye development and abnormality in shape and size.

c Presence of no, one or more than two otoliths per sacculus, as well as reduction and enlargement of otoliths and/or sacculi (otic vesicles).

d Tail malformation was recorded when the tail was bent, twisted or shorter than to control embryos as assessed by optical comparison.

e Growth retardation was recorded by comparing with the control embryos in development or size (before hatching, at 24 and 48 hpf) or in a body length (after hatching, at and onwards 72 hpf) using by optical comparison using a inverted microscope (CKX41; Olympus).

**Table A2:** The antifungal activity of standard nystatin as MIC values against *Candida* spp., in µg/ml

Organism	MIC µg/ml
*C. albicans*	1
*C. krusei*	2
*C. parapsilosis*	0.125
*C. glabrata*	2

Abbreviations: MIC, minimal inhibitory concentration.

**Table A3:** Toxicological parameters derived from the concentration‐response curves for the assessment of toxicity and anti‐angiogenic potential of staurosporine in comparison to clinically used sunitinib‐malate

					EC_50_	
	LC_50_	ECx_50_	IC_50_	Tw	ISVs	SIVs
Staurosporine	45.74 (97.88)	42.01 (89.9)	1 2.14	46	2.24 (4.79)	4.00 (8.56)
Sunitinib^a^	575.2 (1.08)	383.4 (0.72)	266.3 (0.50)	2.16	937.3 (1.76)	564.5 (1.06)

The values given for staurosporine and sunitinib are in ng/mL and µg/mL, respectively, while those in brackets are in nM and µM, respectively.

LC_50_ – the concentration inducing the lethal effect of 50% embryos. ECx_50_ – the concentration affecting 50% embryos (survival and developmental defects). IC_50_ values ‐ the concentration upon which 50% of embryos displayed anti‐angiogenic phenotype. EC_50_ (SIVs and ISVs) ‐ the effective concentration resulting in 50% decrease of SIVs basket or ISVs length compared to the DMF‐treated control group. Tw‐ the ratio between LC_50_ and IC_50_ values.

a The data for sunitinib‐malate have previously been reported by our group (Pavic et al. *Jour Inorg Biochem*, 174 (2017), 156–168).
